# Supplementary material for: Proteo-genomics of soluble TREM2 in cerebrospinal fluid provides novel insights and identifies novel modulators for Alzheimer’s disease
Source: Mol Neurodegener. 2024 Jan 3;19:1. doi: 10.1186/s13024-023-00687-4 (PMC10763080; doi:10.1186/s13024-023-00687-4)
Supplement: Supplementary file 1 — Additional file 1: Fig. S1. Principal component plots of C2 vs C1 using HapMap Phase II as reference. Fig. S2. Quantile-quantile plot (Q-Q plot) of GWAS for CSF sTREM2 in European individuals (EUR-GWAS). Fig. S3. Manhattan plot and quantile-quantile plots (Q-Q plots) of GWAS for CSF sTREM2 in non-European individuals (nonEUR-GWAS) and meta-analyses of EUR GWAS and nonEUR GWAS. Fig. S4. Scatterplot of effect size across GWAS analyses at four index variants. Fig. S5. Association results of CSF sTREM2 at chromosome 11. Fig. S6. Locus plots of rs12664332 at chromosome 6 for CSF sTREM2 and AD. Fig. S7. Experimental design, representative western blots of TGFBR2 and RBMS3 overexpression and knockdown of TGFBR2 in PBMC-derived macrophages presented in Figure 4. Fig. S8. Box plots of CSF sTREM2 Z Score by APOE ε4 dosage for EURs A), and non-EURs B). Fig. S9. UCSC genome browser visualization of brain cell type specific ATAC-seq, H3K27ac ChiP-seq, H3K4me3 ChiP-seq and PLAC-seq loops at the chr 19 APOE locus. Fig. S10. Experimental design and representative western blots of NECTIN2 overexpression in PBMC-derived macrophages presented in Figure 5 Fig. S11. NECTIN2 knock-down experiments. Quantifications of intracellular NECTIN2 protein levels. The cells were transduced with NECTIN2 shRNAs using MOI of 1 in 6 independent batches (A, B, C, D, E and F) and MOI of 2 in 4 independent batches (H, I, J and K). Each batch includes 3-4 wells transduced with control shRNA and 3 wells transduced with target NECTIN2 shRNAs B and C. Fig. S12. Dot plots and circular plots of proteins associated with rs72918674 at chromosome 11 and rs11666329 at chromosome 19. Fig. S13. Scatter plots of CSF sTREM2 measured using MSD vs SomaScan in Knight ADRC. Fig. S14. Box plots of CSF sTREM2 Z Score by ethnicity and sex. [file 13024_2023_687_MOESM1_ESM.docx]

**Proteo-genomics of soluble TREM2 in cerebrospinal fluid provides novel insights and identifies novel modulators for Alzheimer’s disease**

Lihua Wang^1,2,*^, Niko-Petteri Nykänen^1,2,*^, Daniel Western^1,2^, Priyanka Gorijala^1,2^, Jigyasha Timsina^1,2^, Fuhai Li^3^, Zhaohua Wang^1,2^, Muhammad Ali^1,2^, Chengran Yang^1,2^, Menghan Liu^1,2^, William Brock^1,2^, Marta Marquié^4,5^, Mercè Boada^4,5^, Ignacio Alvarez^6^, Miquel Aguilar^6^, Pau Pastor^7^, Agustín Ruiz^4,5^, Raquel Puerta^4,5^, Adelina Orellana^4,5^, Jarod Rutledge^8^, Hamilton Oh^8^, Michael D Greicius^8^, Yann Le Guen^8^, Richard J. Perrin^9^, Tony Wyss-Coray^8^, Angela Jefferson^10^, Timothy J. Hohman^10^, Neill Graff-Radford^11^, Hiroshi Mori^12^, Alison Goate^13^, Johannes Levin^14^, Yun Ju Sung^1,2,15,^^¶^, Carlos Cruchaga^1,2,16,17,¶^

**^Supplementary figures^**


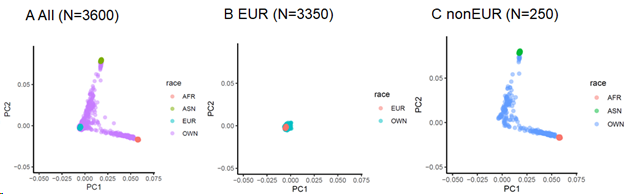


**Fig. S1 Principal component plots of C2 vs C1 using HapMap Phase II as reference. A**) PCA plots for all samples (N=3600). **B**) PCA plots for EURs (N=3350). **C**) PCA plots for nonEURs (N=250). AFR indicates Africans; ASN denotes Asian; EUR indicates Europeans.


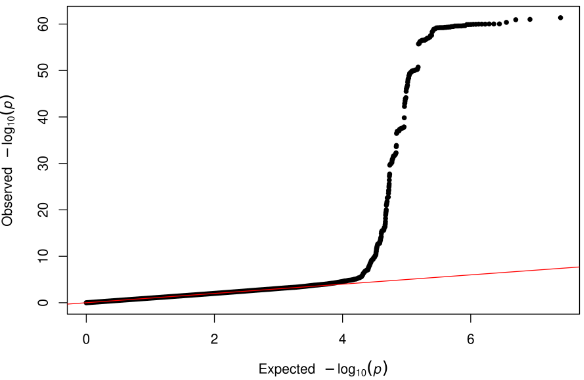


**Fig. S2 Quantile-quantile plot (Q-Q plot) of GWAS for CSF sTREM2 in European individuals (EUR-GWAS)**.

Q-Q plot of EUR-GWAS: P values are two-sided raw P values estimated from a linear additive model. Y-axis represents observed –log10(p) and the x-axis depicts the expected –log10(p).


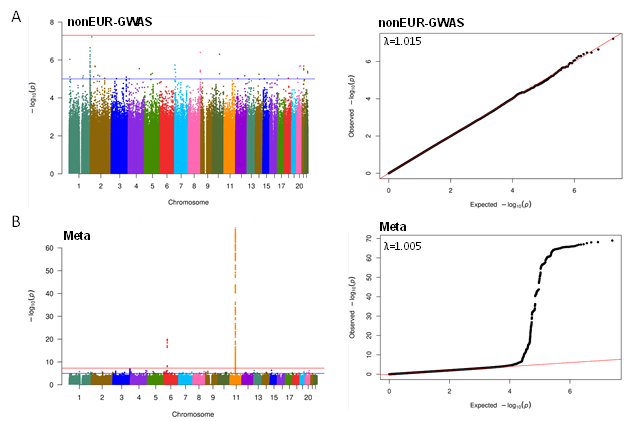


**Fig. S3 Manhattan plot and quantile-quantile plots (Q-Q plots) of GWAS for CSF sTREM2 in non-European individuals (nonEUR-GWAS) and meta-analyses of EUR GWAS and nonEUR GWAS.**

**A**) Left panel is Manhattan plots of nonEUR-GWAS. P values are two-sided raw P values estimated from a linear additive model. The blue solid horizontal line denotes the genome-wide significance level (P = 5×10^-8^), and the red solid horizontal line represents the suggestive significance level (P = 1×10^-6^). X-axis depicts genomic coordinates by chromosome number and y-axis denotes the negative log10-transformed P value for each genetic variant. Right panel is the Q-Q plot of nonEUR_GWAS. Y-axis represents observed -log10(p) and x-axis depicts the expected -log10(p). **B**) Left panel is Manhattan plots of meta-analyses of EUR-GWAS and nonEUR-GWAS for CSF sTREM2. P values are two-sided raw P values estimated from a linear additive model.


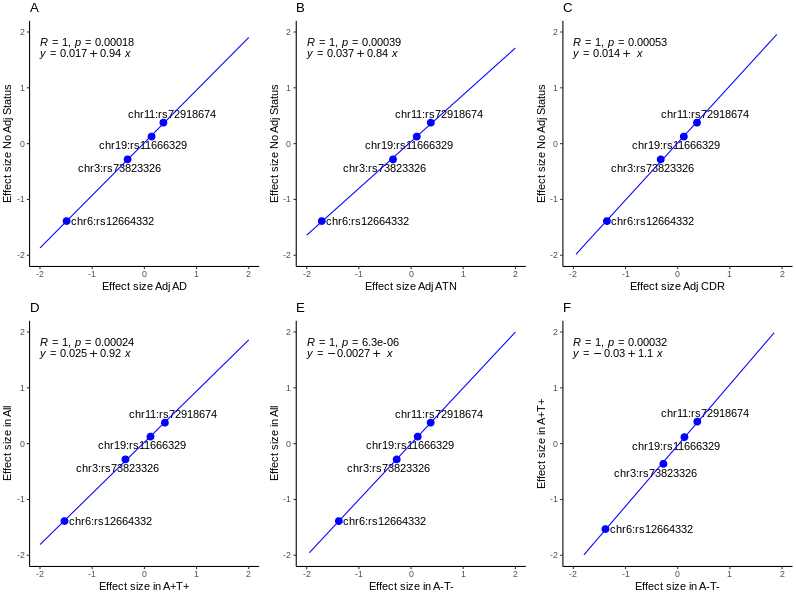


**Fig. S4 Scatterplot of effect size across GWAS analyses at** **four index variants. A)** GWAS with and without adjusting for AD status. **B)** GWAS with and without AT classification. **C)** GWAS with and without adjusting for CDR. **D)** GWAS in all individuals vs. GWAS in biomarker positive (A+T+). **E)** GWAS in all vs GWAS in biomarker negative (A-T-). **F)** GWAS in biomarker positive (A+T+) vs. in biomarker negative (A-T). AD: Alzheimer’s disease; CDR: Clinical Dementia Rating.


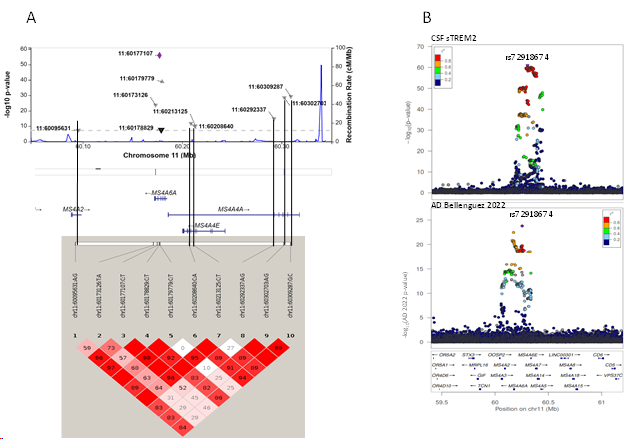


**Fig. S5 Association results of CSF sTREM2 at chromosome 11.**

**A)** LocusZoom plots at chromosome 11 for 10 SNPs (upper panel) and linkage disequilibrium (LD) heatmap of these 10 SNPs based on R2 (the square of the correlation coefficient) estimated using Haploview 4.2 (lower panel); **B)** LocusZoom plots at chromosome 11 for GWAS of CSF sTREM2 and GWAS of Alzheimer’s disease (AD) in 2022. X-axis depicts genomic coordinates at chromosome 11 and y-axis denotes the negative log10-transformed P value for each genetic variant.

**
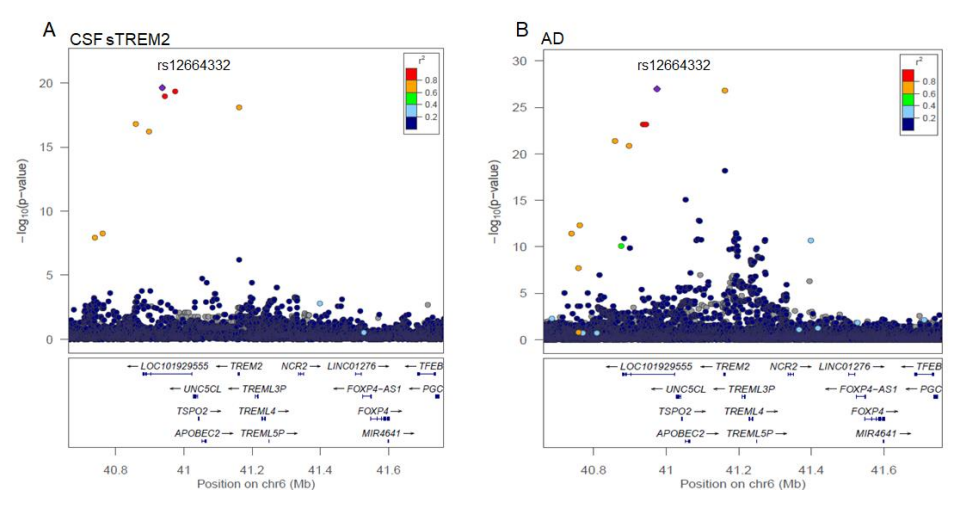
**

**Fig. S6 Locus plots of rs12664332 at chromosome 6 for CSF sTREM2 and AD**

**A)** LocusZoom plots at chromosome 6 for GWAS of CSF sTREM2. **B)** LocusZoom plots at chromosome 6 for GWAS of AD in 2022. The X-axis depicts genomic coordinates at chromosome 6 and the y-axis denotes the negative log10-transformed P value for each genetic variant.


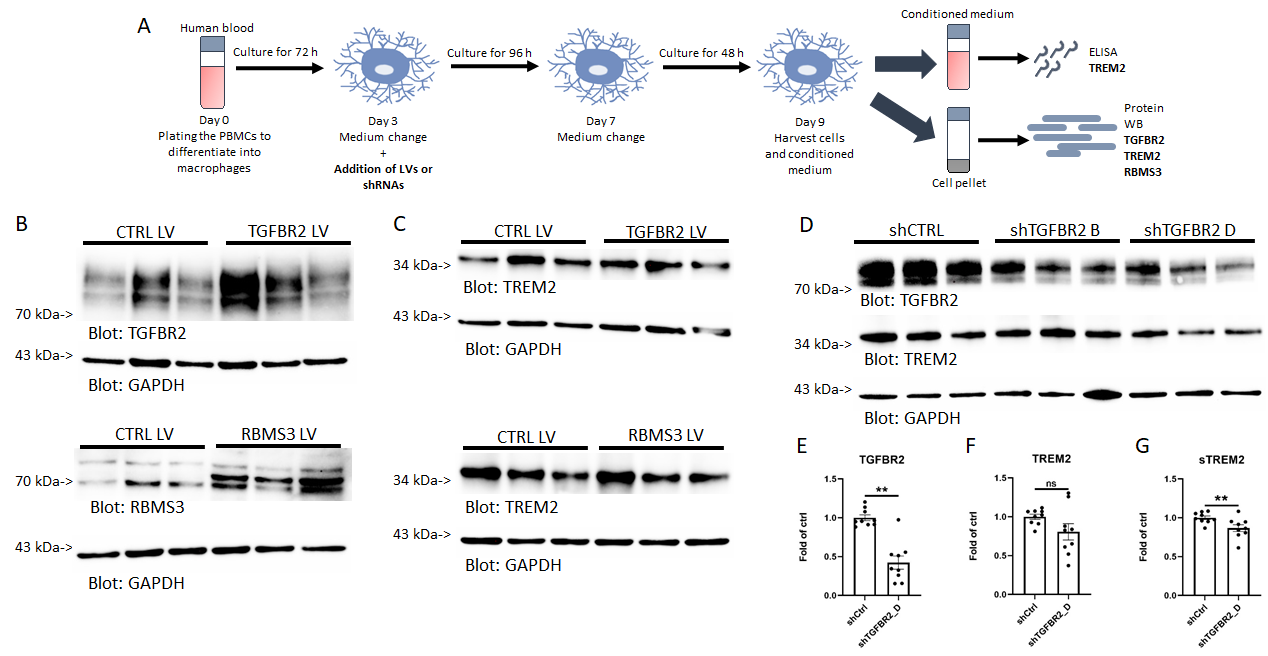


**Fig. S7 Experimental design, representative western blots of *TGFBR2* and *RBMS3* overexpression and knockdown of *TGFBR2* in PBMC-derived macrophages presented in Figure 4**

**A)** Experimental design for PBMC-derived macrophages. **B)** Intracellular TGFBR2 and RBMS3 protein levels and **C)** TREM2 levels upon TGFBR2 and RBMS3 overexpression. **D)** Representative western blot upon TGFBR2 knock down. Quantification of intracellular **E)** TGFBR2, **F)** intracellular TREM2 and G) extracellular sTREM2 levels upon *TGFBR2* knockdown using TGFBR2_D shRNA. n = 9 from 3 independent experiments. ns: not significant, ** p < 0.01. Results are shown in mean ± SEM.

**Fig. S8 Box plots of CSF sTREM2 Z Score by APOE *ε4* dosage for EURs A), and non-EURs B).** Non *APOE* 4 indicates no copy: ε2/ε2, ε2/ 3, and ε3/ε3; *APOE* 4X denotes one copy of ε4 (ε2/ε4 and ε3/ε4), and *APOE* 44 depicts two copies of ε4 (*ε4/ ε4*).


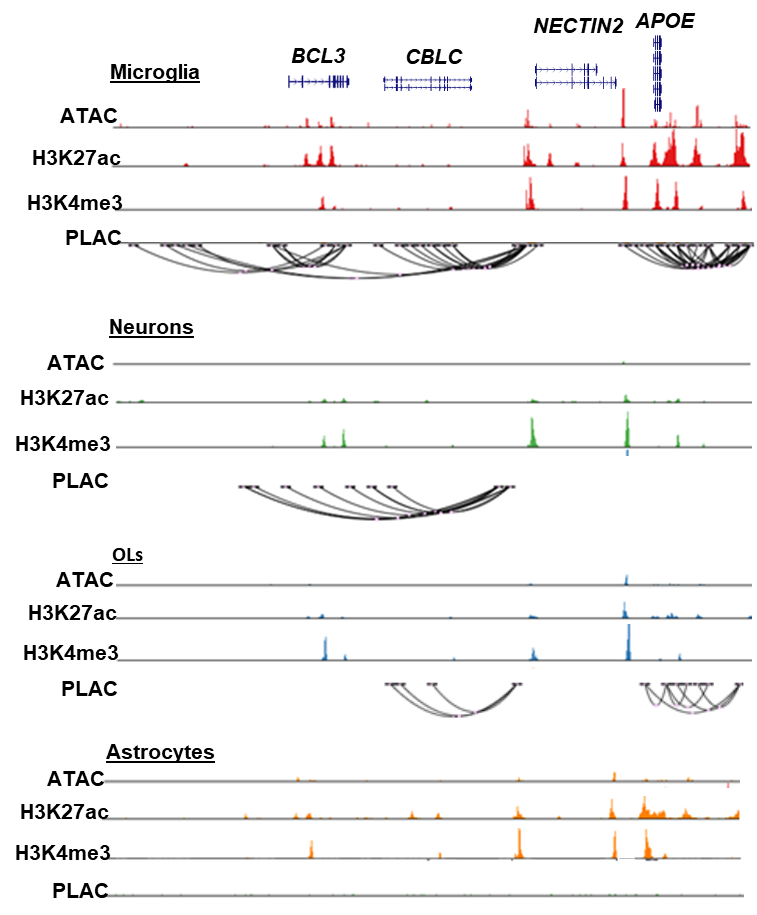


**Fig. S9 UCSC genome browser visualization of brain cell type specific ATAC-seq, H3K27ac ChiP-seq, H3K4me3 ChiP-seq and PLAC-seq loops at the chr 19 *APOE* locus.**

Chromatin loops link to promoters of *NECTIN2* to active gene-regulatory region are identified in microglia, neurons and oligodendrocytes (OLs).


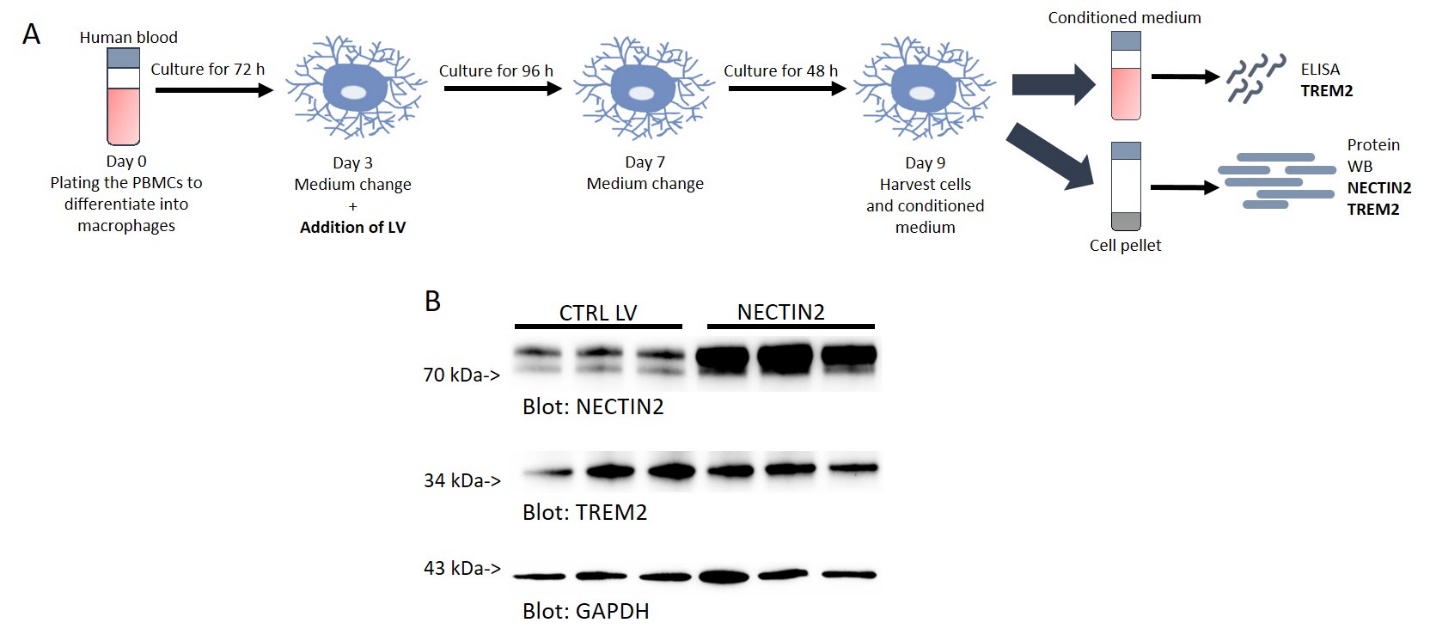


**Fig. S10. Experimental design and representative western blots of *NECTIN2* overexpression in PBMC-derived macrophages presented in Figure 5**

**A)** Experimental design for PBMC-derived macrophages. **B)** Representative western blots of intracellular NECTIN2 and TREM2 protein levels upon *NECTIN2* overexpression.


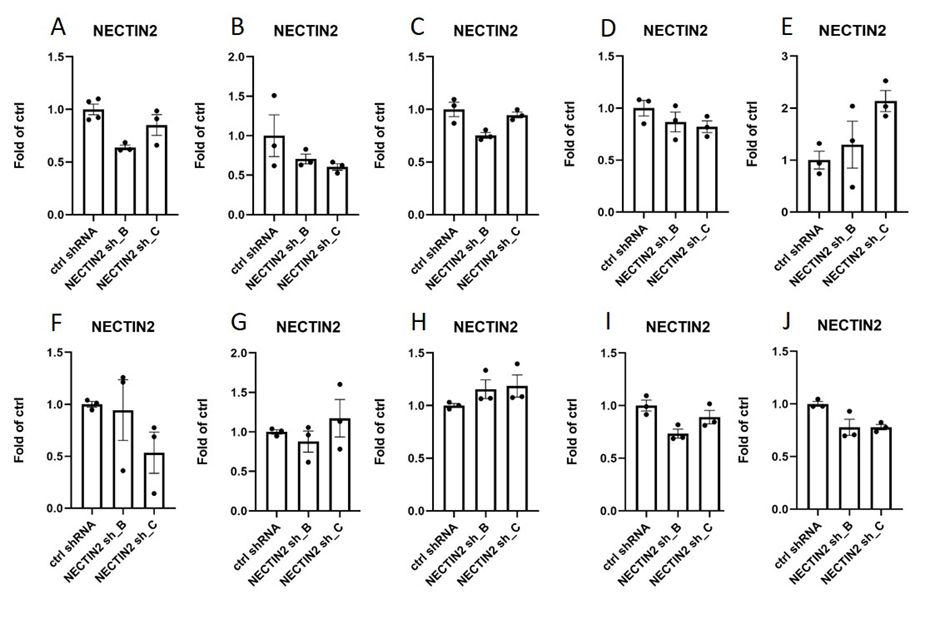


**Fig. S11.** NECTIN2 knock-down experiments. Quantifications of intracellular NECTIN2 protein levels. The cells were transduced with NECTIN2 shRNAs using MOI of 1 in 6 independent batches (A, B, C, D, E and F) and MOI of 2 in 4 independent batches (H, I, J and K). Each batch includes 3-4 wells transduced with control shRNA and 3 wells transduced with target NECTIN2 shRNAs B and C.

**Fig. S12 Dot plots and circular plots of proteins associated with rs72918674 at chromosome 11 and rs11666329 at chromosome 19. A)** Dot plots of –log10(FDR) by proteins for rs72918674 and rs11666329. **B)** Circular plots of proteins associated with rs72918674 and rs11666329. **C)** Tile plots for pathways enriched in 47 proteins.

**Fig. S13 Scatter plots of CSF sTREM2 measured using MSD vs SomaScan in Knight ADRC** (A), SomaScan vs Alamar NULISAseq in Knight ADRC (B), MSD vs Alamar NULISAseq in Knight ADRC (C), and SomaScan vs MSD in DIAN (D).

**Fig. S14 Box plots of CSF sTREM2 Z Score by ethnicity and sex.** EURs_Female: Europeans female; EURs_Male: Euroeans male; non-EURs_Female: non-Europeans female; non-EURs_Male: non-Europeans male
